# A comprehensive examination and analysis of the effectiveness and safety of finerenone for the treatment of diabetic kidney disease: a systematic review and meta-analysis

**DOI:** 10.3389/fendo.2024.1461754

**Published:** 2024-12-20

**Authors:** Jianyu Chen, Jisu Xue, Jiahui Chen, Tingfei Xie, Xiaolu Sui, Yanzi Zhang, Aisha Zhang, Yunpeng Xu, Jihong Chen

**Affiliations:** ^1^ Department of Nephrology, Affiliated Bao’an Hospital of Shenzhen, The Second School of Clinical Medicine, Southern Medical University, Shenzhen, China; ^2^ Department of Nephrology, The Second People’s Hospital of Shenzhen, Shenzhen, China; ^3^ Department of Endocrinology,The People’s Hospital of Baoan Shenzhen, Shenzhen, China; ^4^ Department of Nephrology, The People’s Hospital of Baoan Shenzhen, Shenzhen, China

**Keywords:** diabetic kidney disease, renal function, COVID-19, malignancy, finerenone, meta-analysis

## Abstract

**Objectives:**

The study will evaluate the effectiveness and safety of finerenone in patients diagnosed with diabetic kidney disease (DKD).

**Methods:**

Various databases including PubMed, Sinomed, Web of Science, Embase, Clinical Trials, and Cochrane Library were systematically reviewed for pertinent studies published from the beginning to February 2024.This meta-analysis utilized RevMan 5.3 and Stata 15.1.

**Results:**

The analysis of 4 randomized controlled trials involving 13,943 participants found that finerenone treatment significantly decreased the urine albumin-to-creatinine ratio compared to placebo. Additionally, the risk of COVID-19, cardiovascular events, and estimated glomerular filtration rate(eGFR) reduction of at least 40% were all significantly lower in the finerenone treatment group. However, the finerenone group did experience higher baseline increases in serum potassium levels. The meta-analysis revealed that there was no variation in the likelihood of general negative outcomes (RR 1.00, 95% CI 0.98, 1.01, I^2^ = 0%) and the occurrence of cancers (RR 0.99, 95% CI 0.83, 1.18,I^2^ = 0%) among the two categories.

**Conclusion:**

Our study demonstrates that finerenone has the potential to lower the chances of end-stage kidney disease, renal failure and cardiovascular mortality in individuals with diabetic kidney disease. It is important to monitor for hyperkalemia risk. The administration of finelidone among individuals with diabetic kidney disease may potentially mitigate the susceptibility to contracting COVID-19.

**Systematic review registration:**

https://www.crd.york.ac.uk/prospero/, identifier CRD42024536612.

## 1 Introduction

Diabetes constitutes a severe health threat to the global population, with its incidence on an upward trajectory year by year. The International Diabetes Federation predicts that around 643 million individuals between the ages of 20 and 79 will be diagnosed with diabetes globally by the year 2035.Approximately 40% of people diagnosed with diabetes will develop DKD within 10 to 20 years (1). DKD is the primary factor in the development of advanced kidney disease, characterized by abnormalities in blood sugar regulation, inflammation, oxidative stress, endoplasmic reticulum stress, autophagy, and exosomes. Pathologically, the progression of DKD encompasses mesangial expansion, nodular lesions, and tubulointerstitial fibrosis (2). Despite aggressive treatment with angiotensin-converting enzyme inhibitors(ACEI),angiotensin receptor blocker (ARB) and sodium-dependent glucose transporters 2(SGLT2) inhibitors, the advancement of DKD can be slowed down, but there is still a risk of proteinuria and progression of renal disease (3).

New research has emphasized the crucial importance of excessive activation of the mineralocorticoid receptor (MR) in the advancement of DKD and the related health risks and death rates. By inducing inflammation and fibrosis in the heart, kidneys, and blood vessels, it speeds up the advancement of renal and cardiovascular disease (CVD) (4). Preclinical studies have demonstrated that blocking MR pharmacologically can decrease proteinuria, renal fibrosis, glomerular lesions, and inflammation, while also providing positive effects on cardiovascular health (5). This suggests that mineralocorticoid receptor antagonist(MRA)could be a useful treatment for slowing the progression of chronic kidney disease(CKD).Patients with CKD and chronic heart failure are strongly advised to use steroidal MRA. The initial spironolactone and subsequent eplerenone are among these. Nevertheless, they carry risks of hyperkalemia, declining kidney function, gynecomastia, menstrual irregularities in women, and impotence in men. Therefore, a novel nonsteroidal selective MRA is needed to address the inherent drawbacks of steroidal MRA.

Finerenone is an innovative nonsteroidal MRA exhibiting anti-inflammatory and antifibrotic characteristics. It is a naphthyridine derivative that was synthesized utilizing a dihydropyridine framework and was discovered through the screening of a vast library of compounds (6). It distributes uniformly in the heart and kidneys, possessing a stereostructure and side chains, thus binding more effectively to the MR than spironolactone and eplerenone, exhibiting more pronounced MR antagonism, and providing more significant anti-inflammatory and antifibrotic effects with enhanced protection of cardiac and renal function. Finerenone demonstrates a reduced occurrence of adverse events, particularly hyperkalemia, in comparison to spironolactone. The reduced ability to bind to androgen and progesterone receptors helps to minimize any adverse impacts on sex hormones (7). Preclinical comparative studies have revealed favorable characteristics for the protection of cardiac and renal end-organs. Therefore, finerenone offers a hopeful remedy for the unfulfilled medical need for kidney and heart safeguarding. In 2021, finerenone was approved by the Food and Drug Administration for managing Type 2 Diabetes Mellitus and CKD, with the goal of slowing down the decrease in eGFR and reducing the risk of cardiovascular events. Both the American Heart Association and the American Diabetes Association support the utilization of it (8).

Multiple recent meta-analyses have evaluated the effectiveness of finerenone compared to placebo in improving composite renal function. Although these studies showed consistent advantages for the kidneys, differences were observed in the decrease of eGFR. To provide an accurate assessment of the effectiveness and safety of finerenone in the treatment of DKD, we carried out a comprehensive systematic review and meta-analysis.

## 2 Materials and methods

The meta-analysis was registered on PROSPERO (CRD42024536612) and adhered to the guidelines set forth in the Preferred Reporting Items for Systematic Reviews and Meta-Analyses (PRISMA) statement (9).

### 2.1 Sources of data

A systematic search was conducted across six international databases—PubMed, Sinomed, Web of Science, Embase, ClinicalTrials.gov, and the Cochrane Library—from their inception until December 31, 2023. The search targeted English-language studies that met predefined eligibility criteria. Comprehensive search strategies were developed utilizing Medical Subject Headings (MeSH terms) and relevant keywords, including ‘finerenone’ and ‘diabetic kidney disease’. The search strategy is comprehensively detailed in **Supplementary Table S1** of the **Supplementary Materials**. Furthermore, we conducted a manual search of references, encompassing both research studies and clinical trials, to identify potentially eligible studies. Our search methodology adheres to the PRISMA Guidelines (9). The search strategy is presented in **Supplementary Table S1**.

### 2.2 Inclusion and exclusion criteria

The inclusion criteria were (1):Individuals aged 18 years or older diagnosed with type 2 diabetes and concurrent CKD, characterized by a urinary albumin-creatinine ratio (UACR) of 30 mg/g or higher and an eGFR of 90 ml/min/1.73 m² or less.(2)Follow-up of at least 12 weeks;(3)Administering finerenone orally as a treatment at any dose;(4)Placebo used in the control group;(5)Including at least one important outcome in the report;(6)English-language published randomized controlled trials (RCTs).

We excluded studies that met any of the following criteria:(1)other forms of diabetes;(2) patients receiving renal replacement therapy;(3)glycated hemoglobin >12% at the run-in visit or the screening visit;(4) animal experiments and *in vitro* studies;(5)at the time of screening, the patient’s serum potassium level exceeded 4.8mmol/L;(6)meta-analyses, case reports, non-randomized trials, letters, or studies lacking control or placebo groups.

To screen and select studies for this meta-analysis, PICOS criteria were used,.

P: DKD patients.

I: Finerenone.

C: Control.

O: (a). a sustained decrease of at least 40% in the eGFR from baseline,(b).alterations in UACR from the starting point,(c). any negative effects linked to the intervention,(d). occurrences of cancer,(e)cases of COVID-19.

S:The study only included RCTs.

### 2.3 Study selection

The two authors (JC, JX) independently conducted a comprehensive review of the study selection process, encompassing the initial screening of titles and abstracts, as well as the detailed examination of full texts. They employed standardized tables for data extraction. Potentially relevant materials were identified through a manual review of reference lists and unpublished data from clinical trial websites. Any discrepancies were resolved through discussion with the third author (TX) to achieve consensus.

### 2.4 Data extraction

Two authors (JC, JX) collected significant data. In the event of any discrepancies, a third-party reviewer (TX) will be consulted to address and resolve the issue. The data extracted included details of the study, patient information (such as inclusion criteria, average age, baseline eGFR, and baseline UACR), and reported results (including changes in UACR from baseline, number of patients with ≥40% decrease in eGFR from baseline, overall adverse events, occurrences of malignancies, COVID-19 cases, and alterations in serum potassium levels from baseline).

### 2.5 Methodological quality assessment

The quality of the included studies was assessed using the revised Cochrane Risk of Bias tool (ROB 2.0) outlined in the Cochrane Handbook for Systematic Reviews of Interventions (10). The items evaluated include: the randomization process, deviations from the intended experimental protocols, missing outcome data, the measurement accuracy of the outcomes, and the selection criteria for the reported results. Each item was categorized as having a high risk of bias, unclear risk of bias, or low risk of bias. The collective evidence was utilized to evaluate the study’s risk of bias. Two reviewers (JC and JX) conducted the assessment, and any discrepancies were adjudicated by a third reviewer (TX).

### 2.6 Statistical analysis

Data analysis was combined using Reviewer Manager 5.3 (Copenhagen: Nordic Cochrane Centre, The Cochrane Collaboration, 2014). A hazard ratio (RR) with a 95% confidence interval (CI) was used in the study to show dichotomous outcomes, while a mean difference (MD) with a 95% CI was used for continuous outcomes (change from the starting point to a specific follow-up time). I^2^ statistics are used to examine heterogeneity in included studies (11), and the Cochrane Manual recommends I^2^ values of 25%, 50%, and 75% to indicate low, medium, and high heterogeneity, respectively (12). When the heterogeneity test result (I^2^) of the included studies was less than 50%, fixed-effect model meta-analysis was used. Alternatively, when the heterogeneity test results of the included studies were greater than 50%, the random effects model was used for meta-analysis. Stata 15.1 software was used to conduct sensitivity analysis of the efficacy outcome indicators reported in all four studies included in this paper to evaluate the stability of the results. In consideration of aldosterone escape and the effect of treatment course on UACR, subgroup analysis was performed for 4 studies, which were classified as finerenone treatment course < 3 months and finerenone treatment course ≥3 months.

### 2.7 Patient approval and clearance from the ethical committee

This article relies on past studies and does not include any new research on human or animal subjects conducted by the authors. Therefore, the consent of the patient or the ethics committee is not required. The study protocols received approval from the ethics committees at each participating site.

## 3 Results

### 3.1 Literature search results

**Figure 1:**
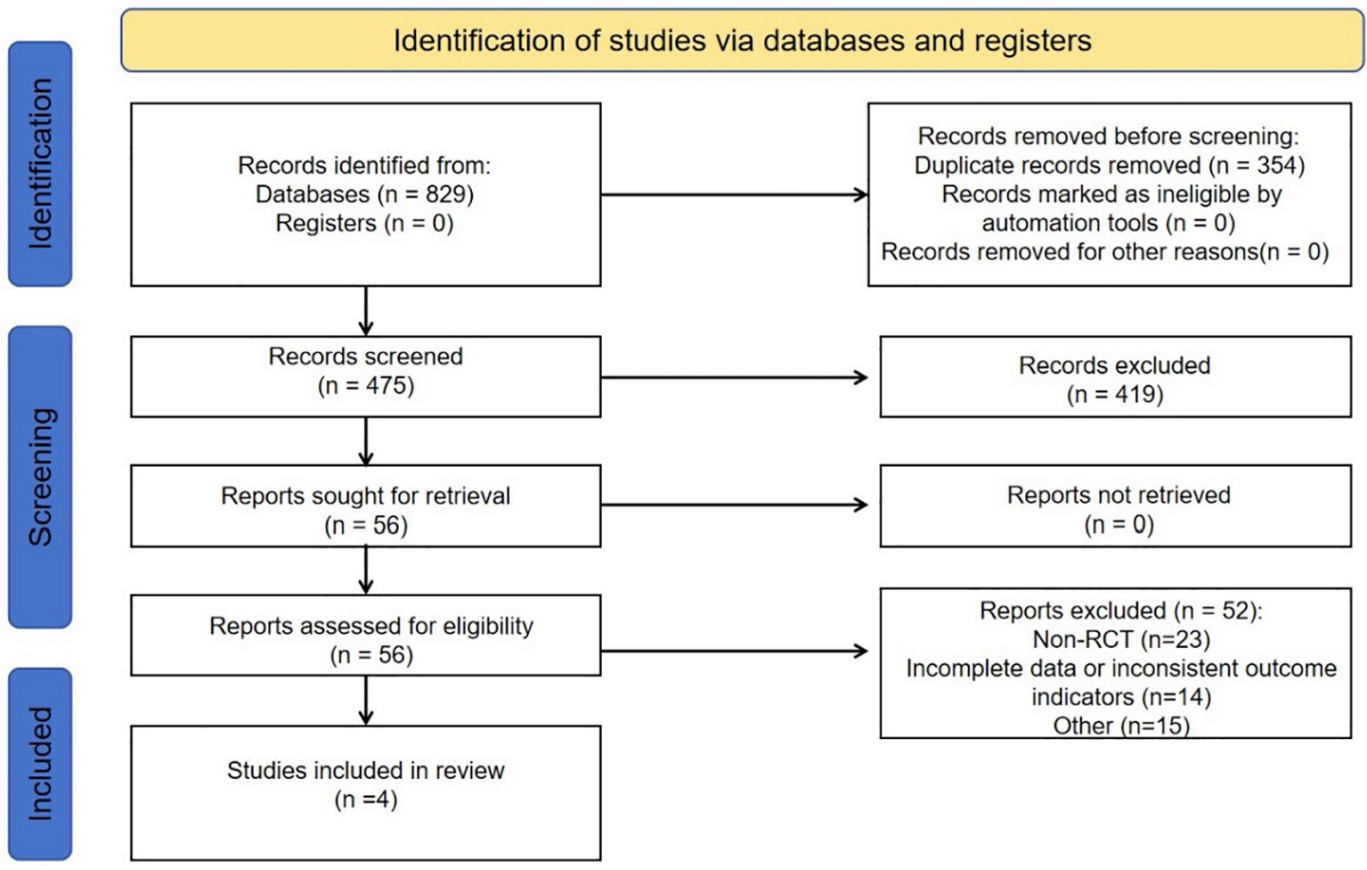
Flowchart elaborating on study retrieval and inclusion in the meta-analysis. RCT, randomized controlled trial.

After initial searching, a total of 829 articles were identified. After reviewing titles, abstracts, and full texts, we selected 56 studies for thorough assessment, ultimately including four qualifying studies in our analysis. There were no additional studies identified through manual search. The screening process is outlined in **Figure 1**.

### 3.2 Research characteristics

The meta-analysis included 4 studies(Pitt et al., 2021;Katayama et al., 2017;Bakris et al., 2020; Bakris et al., 2015)with a total of 13,943 participants(finerenone n=7330;placebo n=6613).Every study documented the positive effects and negative reactions of finerenone in individuals with diabetic kidney disease, with the patient population in each trial varying from 96 to 7352. Further information on the studies can be found in **Table 1**.

**Table 1:** Baseline characteristics of the citations included for analysis.

Study	Country	Sponsorship	Conflicts Of Interest	Total Participants		Age(years)		Follow-up time	1.Changes in UACR from baseline		2.patients with ≥40% decrease in the eGFR from baseline		3.Changes in Serum Potassium (mmol/l)from baseline		4.Cardiovascular event		5.Total adverse events		6.COVID-19 Events		7.malignant neoplasms	
				Finerenone	Placebo	Finerenone	Placebo		Finerenone	Placebo	Finerenone	Placebo	Finerenone	Placebo	Finerenone	Placebo	Finerenone	Placebo	Finerenone	Placebo	Finerenone	Placebo
Bakris (14)	Multicenter	Bayer	YES	727	94	64.3±9.2	63.3±8.7	90days	0.74±0.55	0.94±0.68	7/715	2/93	0.15±0.39	-0.004±0.44	3/727	1/94	386/727	47/94	NA	NA	3/727	0/94
Pitt (11)	Multicenter	Bayer	YES	3686	3666	64.1±9.7	64.1±10.0	3.4years	0.62±0.45	0.92±0.75	350/3686	395/3666	0.18±0.47	0.03±0.46	458/3686	519/3666	3134/3683	3129/3658	83/3683	115/3658	149/3863	154/3658
Katayama (12)	Japan	Bayer	Not Mentioned	84	12	62.4±9.8	66.8±9.0	90days	0.86±0.42	1.06±0.53	0/83	0/12	0.027±0.336	-0.075±0.182	4/84	0/12	40/84	6/12	NA	NA	NA	NA
Bakris (13)	Multicenter	Bayer	Yes	2833	2841	65.4±8.9	65.7±9.2	2.6 Years	0.65±0.53	0.95±0.72	479/2833	577/2841	0.25±12.81	0.02±12.81	367/2833	420/2841	2468/2827	2478/2831	3/2827	3/2831	103/2827	92/2831

N/A, not applicable; GFR, glomerular filtration rate; RCT, randomized controlled trial.

2. patients with ≥40% decrease.

### 3.3 Quality of evidence

**Figure 2:**
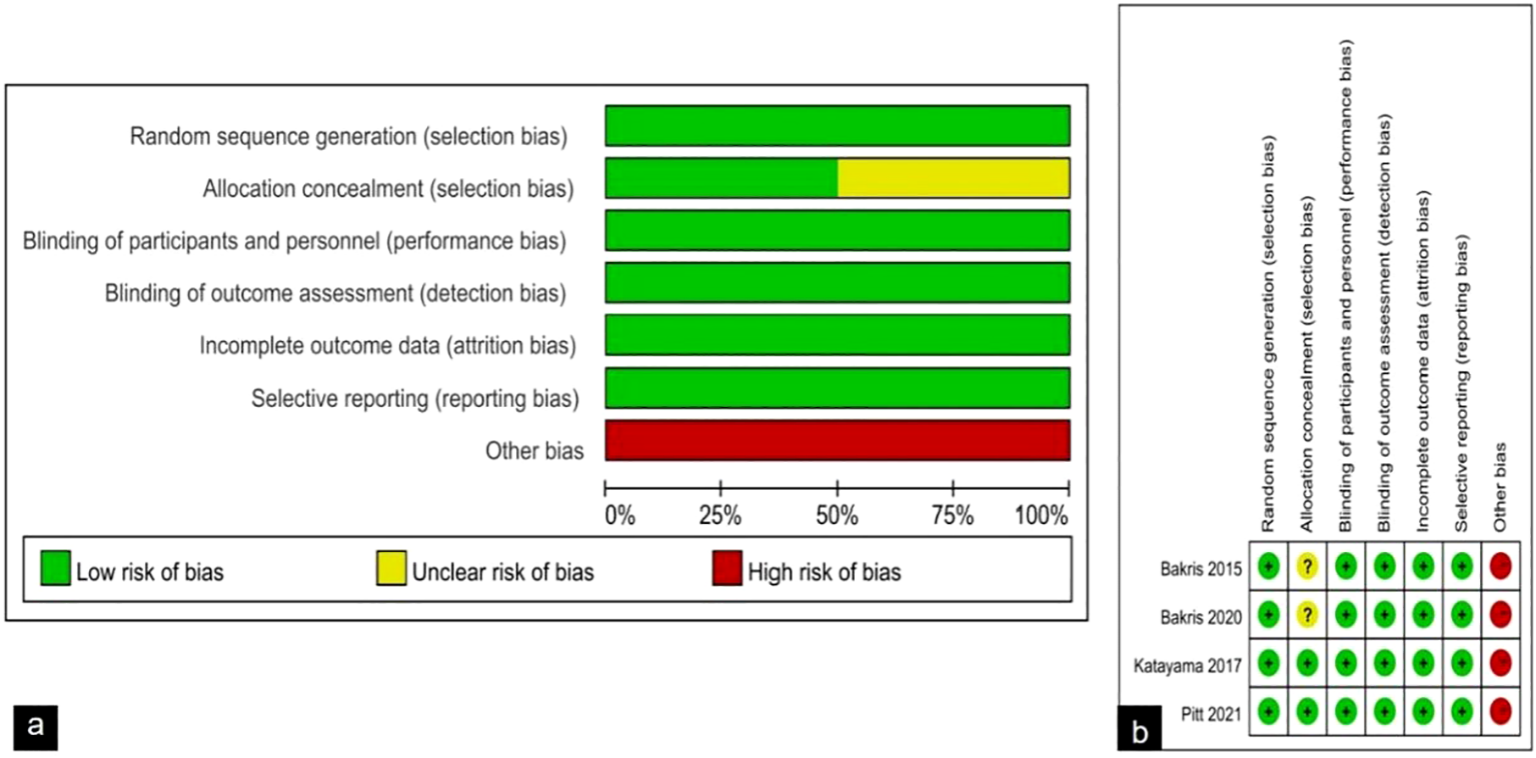
**(A)** Risk of bias graph: review authors' judgements about each risk of bias item presented as percentages across all included studies; **(B)** Risk of bias summary: review authors' judgements about each risk of bias item for each included study.

The risk of bias is discussed using the Cochrane Handbook for Systematic reviews of interventions, and overall the included trials showed a low to moderate risk of bias. The results of bias risk assessment for all included studies are shown in **Figure 2**. Regarding bias in randomization, all four studies specifically described the use of computer random assignment. All four RCTS were considered low-risk in terms of selective reporting, blinding of participants and personnel, and bias due to blinding in outcome assessment. For bias associated with allocation concealment, 2 studies were considered at risk of ambiguity, while 2 studies were classified as low risk. The four RCTS all reported incomplete data such as loss of follow-up and withdrawal, but did not affect the study results, and all reported the study results completely, so they were not considered as sources of bias. Factors contributing to the bias include: 1) The four randomized controlled trials included in this study were all funded by Bayer, the manufacturer of finerenone, where there may be potential risks, and 2) the two studies did not describe specific methods of allocation concealment.

### 3.4 Sensitivity analysis and publication bias

**Figure 3:**
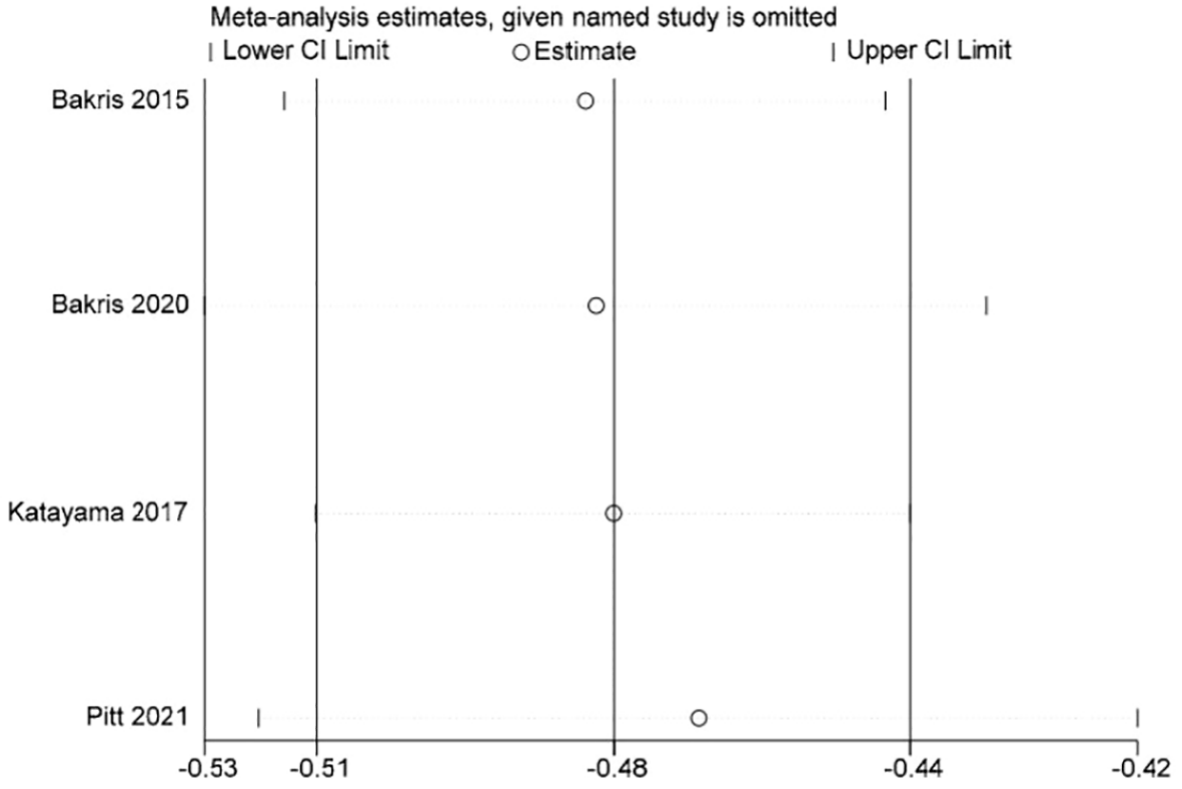
Sensitivity analysis diagram of UACR.

**Figure 4:**
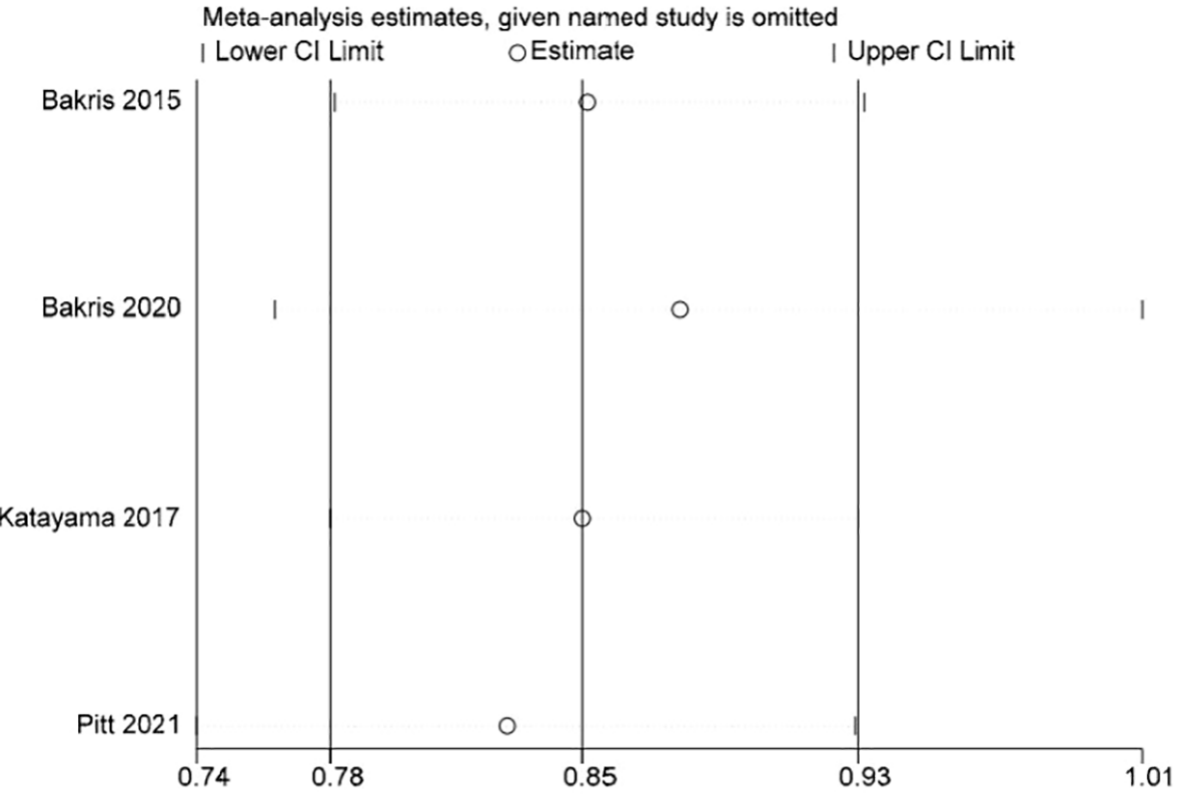
Sensitive analysis graph of event incidence of eGFR decline ≥40%.

**Figure 5:**
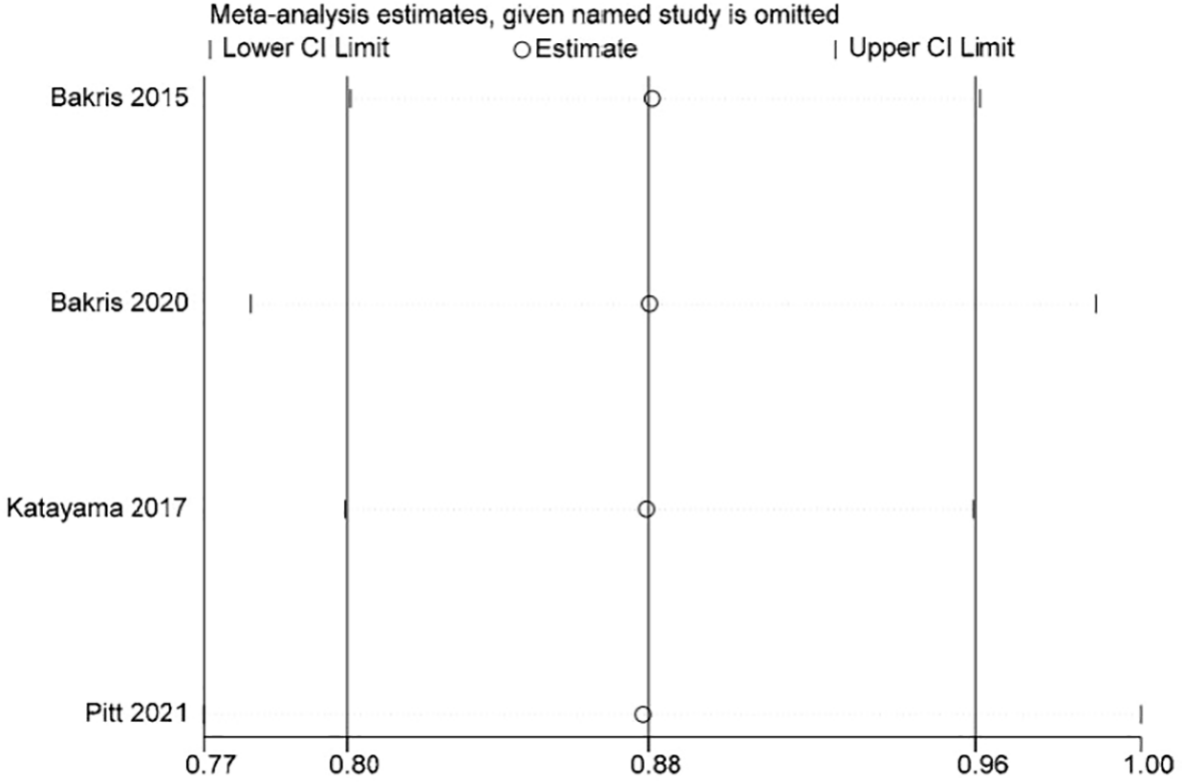
Sensitivity analysis chart of cardiovascular event incidence.

Stata 15.1 software was used to conduct sensitivity analysis of the efficacy outcome indicators reported in all four studies included in this paper, and the evaluation results were stable (**Figures 3**–**5**).

### 3.5 Report outcomes

#### 3.5.1 UACR

**Figure 6:**
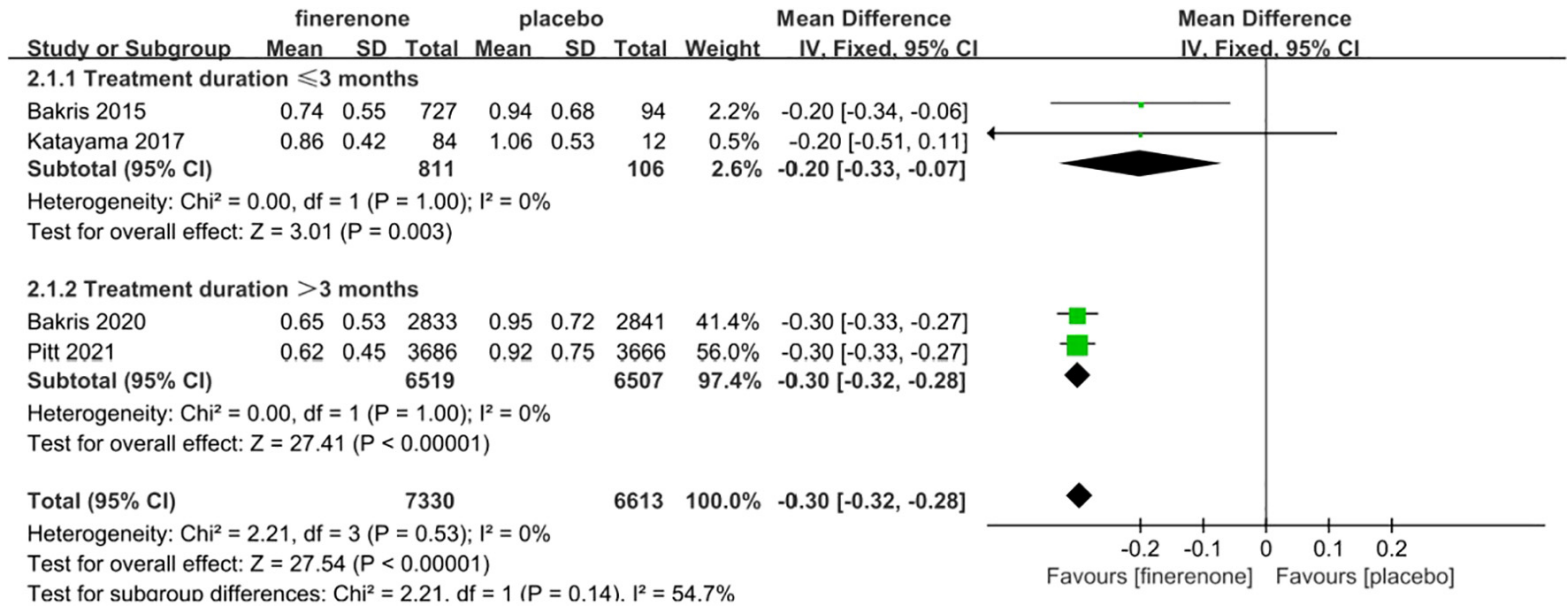
Meta-analysis results of the effect of finerenone versus placebo on UACR.

Four studies reported UACR, a total of 13,943 patients were included: 7330 patients in the finerenone group and 6613 patients in the Placebo group (13–16). A fixed-effect model was utilized for analyzing the change in UACR from baseline to follow-up time, as there was minimal variability in the extracted data (P=0.53, I^2^ = 0%).The data indicated that the finerenone group had a significantly decreased change in UACR from baseline compared to the placebo group, with a mean difference of (MD: -0.30; 95%CI [-0.32, -0.28]; P < 0.00001; I^2^ = 0%) (**Figure 6**), showing a statistically significant distinction. Taking into account aldosterone escape and the effect of treatment course on UACR, subgroup analysis of 4 studies showed that: In the treatment group ≤3 months, MD=-0.20, 95%CI(-0.33, -0.07), P=0.003,In the treatment group > 3 months, MD=-0.30, 95%CI(-0.32, -0.28), P<0.00001,The results of subgroup analysis showed statistically significant differences, and the results suggested that UACR decreased more significantly with the extension of treatment course.

#### 3.5.2 Reduction in glomerular filtration rate by ≥40%

**Figure 7:**
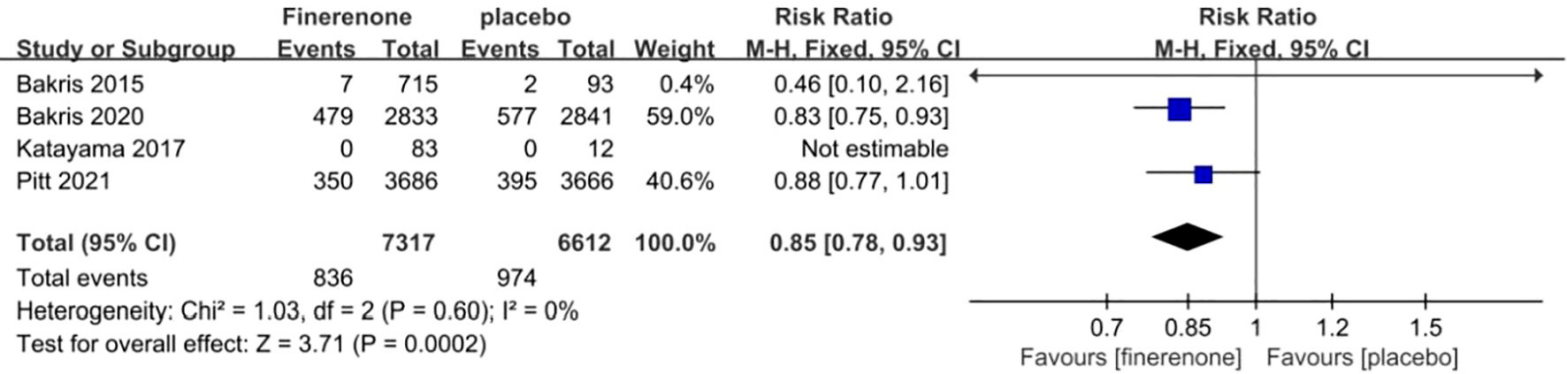
Meta-analysis results of the proportion of patients with ≥40% decrease in the eGFR in finerenone versus placebo patient groups.

The incidence of a reduction in eGFR by ≥40% was documented in all four studies (13–16). The information suggested low heterogeneity in the studies (I^2^ = 0%, P = 0.60), leading to the adoption of a fixed-effect model (RR 0.85; 95%CI [0.78, 0.93]; P = 0.60, I^2^ = 0%) (P < 0.05) **Figure 7**, demonstrating a significant discrepancy. The findings indicate that the finerenone group had a decreased likelihood of eGFR reduction by ≥40% from baseline levels when compared to the placebo group.

#### 3.5.3 Serum potassium concentration

**Figure 8:**
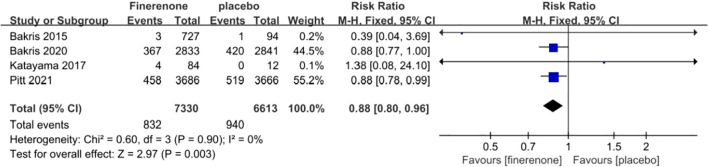
Meta-analysis results of the effect of finerenone versus placebo on cardiovascular events.

In four studies, the difference in serum potassium levels from the initial measurement was compared between the groups receiving finerenone and placebo for patients with DKD. **Figure 8** illustrates that there was minimal variation in the data across the studies (I^2^ = 0%, P = 0.90). The finerenone group showed a greater rise in serum potassium levels at baseline compared to the placebo group (MD 0.15; 95%CI [0.13, 0.17]; P = 0.90, I^2^ = 0%) (P < 0.05).

#### 3.5.4 COVID-19

**Figure 9:**
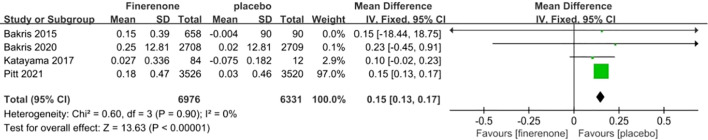
Meta-analysis results of changes in serum potassium concentration between finerenone and placebo patient groups.

Two research studies examined the prevalence of COVID-19, with a combined total of 13,026 participants - 6519 in the finerenone group and 6507 in the Placebo group (13, 15). The studies showed minimal variation (I^2^ = 0%, P = 0.69), leading to the use of a fixed-effect model (RR 0.72; 95%CI [0.55, 0.95]; P = 0.69, I^2^ = 0%) (P < 0.05), indicating a statistically significant distinction. The findings suggest that the occurrence of COVID-19 was reduced in the finerenone group when compared to the placebo group, as shown in **Figure 9**.

#### 3.5.5 Malignancy

**Figure 10:**
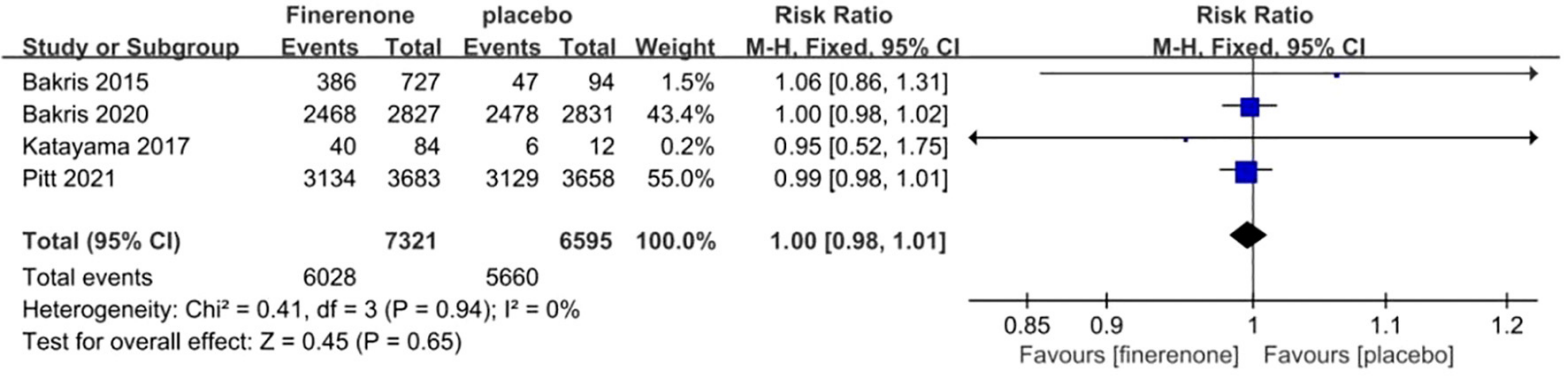
Meta-analysis results of adverse events in finerenone compared to placebo patient groups.

Three studies reported on the incidence of malignancy, a total of 13,847patients were included:7,246patients in the finerenone group and 6,601patients in the Placebo group (13, 15, 16). The studies showed minimal variation (I^2^ = 0%, P = 0.53).Utilizing a fixed-effect model [RR = 0.99, 95%CI (0.83, 1.18); P = 0.53, I^2^ = 0%], (P = 0.92) revealed a lack of statistical significance, suggesting no notable variance in malignancy occurrence between the finerenone and placebo cohorts **Figure 10**.

#### 3.5.6 Cardiovascular adverse events

**Figure 11:**
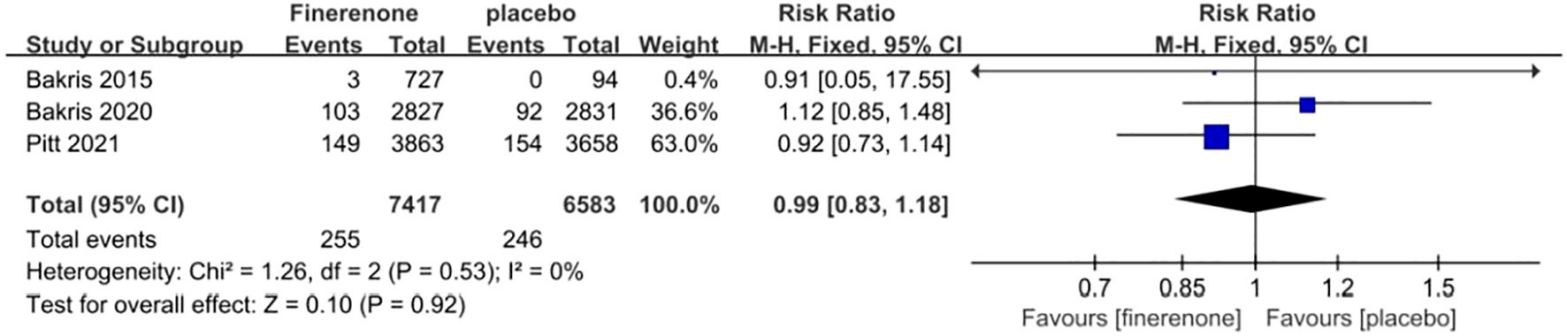
Meta-analysis results of malignant neoplasms in finerenone compared to placebo patient groups.

Each of the four studies examined the occurrence of cardiovascular events (13–16), showing minimal variation among the studies (I^2^ = 0%, P = 0.90).Utilizing a fixed-effect model, the statistical significance of the difference was confirmed with a RR of 0.88 (95%CI [0.80, 0.96]; P = 0.90, I^2^ = 0%) (P < 0.05) **Figure 11**. The findings indicate that there was a notable decrease in cardiovascular events risk in the finerenone group when compared to the placebo group.

#### 3.5.7 Overall adverse events

**Figure 12:**
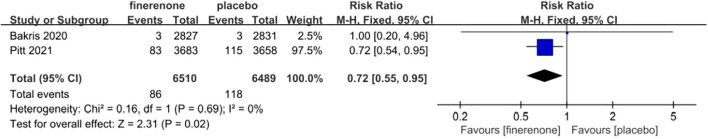
Meta-analysis results of COVID-19 in finerenone compared to placebo patient groups.

Adverse events reported in all RCTs included hyperkalemia, reduced eGFR, arthralgia, constipation, edema, diarrhea, dizziness, and anemia, among others. Each of the four studies provided data on the frequency of general negative occurrences (13–16), showing minimal variation among the studies (I^2^ = 0%, P = 0.65).A fixed-effect model was employed, Finerenone and placebo did not differ statistically significantly in their risk of adverse events. (RR 1.00; 95%CI [0.98, 1.01]; P = 0.94, I^2^ = 0%) (P = 0.65) **Figure 12**.

## 4 Discussion

The link between elevated UACR levels in individuals with DKD and the decline in kidney function, along with the heightened likelihood of cardiovascular issues and heart failure, has been confirmed (17). The meta-analysis showed that patients who received finerenone experienced a substantial decrease in UACR compared to the beginning, along with a marked drop in the percentage of patients with an eGFR decrease of ≥40% from the beginning. This indicates that finerenone may slow the progression of renal dysfunction. Experimental data indicates that the decrease in UACR is due to intricate anti-inflammatory and anti-fibrotic actions resulting from the inhibition of excessive MR activation, with finerenone showing a stronger anti-fibrotic impact in comparison to steroidal MRA (18–20). In another meta-analysis, Fu et al., indicated that while finerenone had a positive effect on reducing UACR, it did not have a beneficial impact on delaying the decline of eGFR (21). In Bakris’ research, it was demonstrated that the finerenone group experienced a slower decrease in eGFR compared to the placebo group after four months; by approximately 26 months, the decline in eGFR was less significant in the finerenone group (15). Our findings suggest that the likelihood of a sustained ≥40% decrease in eGFR was reduced by 15% with the extended use of finerenone, indicating potential kidney benefits for individuals with DKD.

In a heart failure prototype following a heart attack, finerenone demonstrated positive effects on improving both the contraction and relaxation of the left ventricle, as well as cardiac contractility. Furthermore, finerenone has been proven to lower N-terminal pro-b-type natriuretic peptide levels effectively while keeping blood pressure stable. In multiple murine models of vascular injury, finerenone has demonstrated positive vascular effects by promoting vascular integrity and inhibiting detrimental vascular remodeling (22). In patients with both CKD and type 2 diabetes, a follow-up examination of the FIDELIO-DKD study revealed that finerenone was linked to a lower occurrence of new cases of atrial fibrillation or flutter. Moreover, the study showed that finerenone had a positive effect on the overall likelihood of cardiovascular events in these patients, regardless of previous cardiovascular disease (23). These research results are consistent with the findings of our study. The concurrence of CKD and elevated glucose levels significantly heightens the risk of cardiovascular events in patients, surpassing even the risk of ESRD (24, 25). Consequently, it is imperative to focus on cardiovascular benefits for the patient. For this reason, we believe it is necessary to analyze cardiovascular events separately to meet clinicians’ concerns about the risk of cardiovascular events in DKD patients with finelidone. In our study, cardiovascular events were defined as a composite outcome encompassing cardiovascular death, non-fatal myocardial infarction, non-fatal stroke, and hospitalization for heart failure for the purposes of statistical analysis. The findings indicated a significantly lower incidence of cardiovascular events in the finerenone group compared to the placebo group.

Our research suggests that the finerenone treatment group experienced a greater rise in serum potassium levels compared to the placebo group, starting from the initial measurement. However, prior examinations indicate that the finerenone group experienced a notably reduced occurrence of hyperkalemia and average potassium concentration change when compared to the spironolactone group. Hence, the advantages of finerenone should not be overshadowed by the potential risk of hyperkalemia. It is recommended that clinical use includes close monitoring of blood potassium levels and the employment of various methods to minimize the impact of hyperkalemia, such as interruption and dose reduction of finerenone therapy or the addition of potassium-binding agents.

We observed a significant reduction in COVID-19 associated with finerenone compared to placebo, a finding that is quite intriguing and suggests that finerenone may reduce the spread of pulmonary infections to lobar or bronchial consolidation. Pitt’s analysis found that inhibiting the mineralocorticoid receptor with finerenone may protect against pneumonia and COVID-19 in individuals with DKD, as indicated in the literature review (26). Finerenone may decrease the incidence of pneumonia and COVID-19 by affecting ACE2 expression and through its anti-inflammatory and anti-fibrotic properties. Notably, pirfenidone and nintedanib have been approved for the treatment of pulmonary fibrosis. Yet, in rodent models of idiopathic pulmonary fibrosis, finerenone decreased pro-inflammatory and pro-fibrotic indicators in pulmonary homogenates, demonstrating greater effectiveness than pirfenidone and nintedanib. Therefore, the improvement of pulmonary inflammation and fibrosis, the amelioration of right heart pressures and pulmonary congestion, and the upregulation of ACE2 expression are various factors that could lead to an improved pulmonary environment, making the spread of pulmonary infections and inflammation less likely (27).Additionally, the antagonistic impact of MRA on mineralocorticoid receptors in monocytes and macrophages may impede the infiltration and aggregation of activated macrophages, thereby mitigating COVID-19-induced pulmonary tissue injury (28–30).

The correlation between Type 2 diabetes and an increased vulnerability to cancer is believed to be linked to elevated levels of blood sugar, insulin, and body weight. Sustained increase in natural insulin and/or Insulin-like growth factor 1 levels could activate cell division signaling, promoting the growth and spread of tumors (27). Prior studies have indicated that naphthyridines found in nature have qualities that can fight infections, hinder cancer development, and regulate the immune system. The production of naphthyridine variations also shows anti-cancer, antibacterial, anti-inflammatory, antioxidant, and immune system-modulating impacts (31, 32). Finerenone and its metabolites belong to the class of naphthyridine derivatives. Our research indicates that there is no significant variance in the occurrence of cancer between the finerenone cohort and the placebo cohort. This is consistent with past studies on the pharmacological actions of naphthyridine derivatives.

Findings from a study conducted by Yue Du et al. indicate that, in comparison to placebo treatment, finerenone does not elevate the general risk of tumors but is linked to a heightened risk of urological malignancies (33). The underlying reasons for this association, whether it stems from the drug’s excretion pathways or other unidentified pharmacological effects of finerenone and its metabolites, necessitate further investigation.

Our study has some advantages. First, to our knowledge, this is the first meta-analysis to focus on the incidence of COVID-19 in DKD patients treated with finerenone. In the future, we hope to explore more about the mechanism by which finerenone reduces the incidence of COVID-19 in DKD patients. Secondly, considering the effects of aldosterone escape and the length of treatment of finerenone on UACR in DKD patients, we conducted subgroup analysis of 4 studies, and the results showed that UACR decreased more significantly with the extension of treatment.

Our study has several limitations. First, we did not analyze the efficacy and safety of different doses of finerenone. Second, the scope of this meta-analysis was limited by the number of studies included, in particular two Phase II and two Phase III trials. In addition, the FIDELIO-DKD study reported a limited number of COVID-19 cases due to the choice of study time period.

## 5 Conclusions

The results of our study indicate that finerenone can effectively decrease the UACR and potentially provide cardiorenal protection for individuals with DKD. However, finerenone may cause an increase in serum potassium, suggesting that close monitoring of potassium levels or concomitant use of potassium-lowering medication should be considered during clinical application of finerenone. Additionally, the reduction of COVID-19 incidence in DKD patients by finerenone has important clinical implications, and its mechanisms warrant further exploration. Finerenone did not substantially raise the overall likelihood of negative outcomes and cancers. The findings from our research endorse finerenone as a hopeful approach in addressing the advancement of DKD.

## Data Availability

Publicly available datasets were analyzed in this study. This data can be found here: The datasets utilized in this meta-analysis can be obtained by contacting the corresponding author.
