# Supplementary material for: A comprehensive examination and analysis of the effectiveness and safety of finerenone for the treatment of diabetic kidney disease: a systematic review and meta-analysis
Source: Front Endocrinol (Lausanne). 2024 Dec 20;15:1461754. doi: 10.3389/fendo.2024.1461754 (PMC11695223; doi:10.3389/fendo.2024.1461754)
Supplement: Supplementary file 1 [file DataSheet1.docx]

**Supplementary Table S1:Search strategy**

| Database | No. | Search strategy | Results | Time |
| --- | --- | --- | --- | --- |
| Embase | #1 | 'diabetic nephropathy'/exp | 58,146 | 18 Apr 2024 |
|  | #2 | 'diabetic nephropathies':ab,ti OR'diabetic kidney disease"ab,ti OR'diabetic kidney diseases':ab,ti OR'diabetes nephropathy'ab,ti OR  'diabetic nephropathy':ab,ti OR'diabetic glomerulosclerosis':ab,ti OR dkd:ab,ti OR dn:ab,ti OR'renal diabetes'ab,ti OR'diabetic  nephrosis'ab,ti OR 'nephropathies,diabetic:ab,ti OR'nephropathy,diabetic':ab,ti OR"kidney disease,diabetic':ab,ti OR "kidney  diseases,diabeticab,ti OR'glomerulosclerosis,diabeticab,ti OR'intracapillary glomerulosclerosis':ab,ti OR'nodular  glomerulosclerosis':ab,ti OR'glomerulosclerosis, nodular':ab,ti OR "kimmelstiel wilson syndrome':ab,ti OR "kimmelstiel -wilson syndrome':ab,ti OR'syndrome, kimmelstiel-wilson': ab,tiOR "kimmelstiel-wilson disease':ab,ti OR"kimmelstiel wilson disease'ab,ti | 54,955 |  |
|  | #3 | #1 OR#2 | 78,637 |  |
|  | #4 | "finerenone'/exp | 954 |  |
|  | #5 | finerenone:ab,ti OR kerendia:ab,ti OR'bay 94-8862':ab,ti | 591 |  |
|  | #6 | #4 OR#5 | 1,000 |  |
|  | #7 | randomized controlled trial'/exp | 819,863 |  |
|  | #8 | randomized controlled trial':ab,tiOR random:ab,ti OR random*:ab,tiOR placebo:ab,tiOR'controlled clinical trial':ab,ti OR'drug  therapy':ab,ti OR trial:ab,i OR groups:ab,i | 5,731,274 |  |
|  | #9 | #7 OR#8 | 5,808,445 |  |
|  | #10 | #3 AND #6 AND #9 | 180 |  |
| PubMed | #1 | ((("Diabetic Nephropathies"[Mesh]) OR (((((((((((((((((((((((Diabetic Nephropathies[Title/Abstract]) OR (diabetic kidney disease[Title/Abstract])) OR (Diabetic Kidney Diseases[Title/Abstract])) OR (diabetes nephropathy[Title/Abstract])) OR (diabetic nephropathy[Title/Abstract])) OR (DKD[Title/Abstract])) OR (DN[Title/Abstract])) OR (diabetic glomerulosclerosis[Title/Abstract])) OR (renal diabetes[Title/Abstract])) OR (diabetic nephrosis[Title/Abstract])) OR (Nephropathies, Diabetic[Title/Abstract])) OR (Nephropathy, Diabetic[Title/Abstract])) OR (Kidney Disease, Diabetic[Title/Abstract])) OR (Kidney Diseases, Diabetic[Title/Abstract])) OR (Glomerulosclerosis, Diabetic[Title/Abstract])) OR (Intracapillary Glomerulosclerosis[Title/Abstract])) OR (Nodular Glomerulosclerosis[Title/Abstract])) OR (Glomerulosclerosis, Nodular[Title/Abstract])) OR (Kimmelstiel Wilson Syndrome[Title/Abstract])) OR (Kimmelstiel -Wilson syndrome[Title/Abstract])) OR (Syndrome, Kimmelstiel-Wilson[Title/Abstract])) OR (Kimmelstiel-Wilson Disease[Title/Abstract])) OR (Kimmelstiel Wilson Disease[Title/Abstract]))) AND (("finerenone" [Supplementary Concept]) OR (((Finerenone[Title/Abstract]) OR (kerendia[Title/Abstract])) OR (BAY 94-8862[Title/Abstract])))) AND (("Randomized Controlled Trial" [Publication Type]) OR ((((((((randomized controlled trial[Title/Abstract]) OR (random[Title/Abstract])) OR (random*[Title/Abstract])) OR (placebo[Title/Abstract])) OR (controlled clinical trial[Title/Abstract])) OR (drug therapy[Title/Abstract])) OR (Trial[Title/Abstract])) OR (groups[Title/Abstract]))) | 107 | 18 Apr 2024 |
| Cochrane | #1 | MeSH descriptor: [Diabetic Nephropathies] explode all trees | 2,023 | 18 Apr 2024 |
|  | #2 | (Diabetic Nephropathies):ti,ab,kw OR(diabetic kidney disease):ti,ab,kw OR(Diabetic Kidney Diseases):ti,ab,kw OR (diabetes nephropathy):ti,ab,kw OR(diabetic  nephropathy):ti,ab,kw OR(DKD):ti,ab,kw OR(DN):ti,ab,kw OR(diabetic glomerulosclerosis):ti,ab,kw OR(renal diabetes):ti,ab,kw OR(diabetic nephrosis):ti,ab,kw OR  (Nephropathies,Diabetic):ti,ab,kw OR(Nephropathy,Diabetic):ti,ab,kw OR (Kidney Disease,Diabetic):ti,ab,kw OR(Kidney Diseases,Diabetic):ti,ab,kw OR  (Glomerulosclerosis,Diabetic):ti,ab,kw OR(Intracapilary Glomerulosclerosis):ti,ab,kw OR(Nodular Glomerulosclerosis):ti,ab,kw OR(Glomerulosclerosis,  Nodular):ti,ab,kw OR(Kimmelstiel Wison Syndrome):ti,ab,kw OR(Kimmelstiel-Wilson syndrome):ti,ab,kw OR(Syndrome, Kimmelstiel-Wilson):ti,ab,kw OR  (Kimmelstiel-Wilson Disease):ti,ab,kw OR(Kimmelstiel Wilson Disease):ti,ab,kw | 15,561 |  |
|  | #3 | #1OR#2 | 15,561 |  |
|  | #4 | Finerenone | 178 |  |
|  | #5 | (Finerenone):ti,ab,kw OR(kerendia):ti,ab,kw OR(BAY 948862):ti,ab,kw | 180 |  |
|  | #6 | #4 OR#5 | 181 |  |
|  | #7 | MeSH descriptor: [Randomized Controlled Trial] explode all trees | 37 |  |
|  | #8 | (randomized controlled trial):ti,ab,kw OR(random):ti,ab,kw OR(random"):ti,ab,kw OR(placebo):ti,ab,kw OR(controlled clinical trial):ti,ab,kw OR(drug therapy):ti,ab,kw  OR(Trial):ti,ab,kw OR(groups):ti,ab,kw | 1,644,004 |  |
|  | #9 | #7OR#8 | 1,644,004 |  |
|  | #10 | #3 AND #6 AND #9 | 137 |  |
| Sinomed | #1 | "糖尿病肾病"[不加权:扩展] | 39,195 | 18 Apr 2024 |
|  | #2 | "糖尿病肾病"[常用字段:智能]OR"糖尿病肾脏疾病"[常用字段:智能]OR"糖尿病性肾病”[常用字段:智能]OR"糖尿病性肾脏疾病”[常用字段:智能]OR"糖尿病性肾小球硬化症”[常用字段:智能]OR"肾小球硬化症”[常用字段:智能]OR"毛细管间性肾小球硬化症”[常用字段:智能]OR"Kimmelstiel"[常用字段:智能]AND"Wilson病”[常用字段:智能]OR"结节性肾小球硬化症”[常用字段:智能] | 49,959 |  |
|  | #3 | (#2)OR(#1) | 49,959 |  |
|  | #4 | 非奈利酮 | 36 |  |
|  | #5 | ”非奈利酮“[常用字段:智能]OR"可申达"[常用字段:智能]OR"BAY948862”[常用字段:智能] | 36 |  |
|  | #6 | (#5)OR(#4) | 36 |  |
|  | #7 | “随机对照试验”[不加权:扩展] | 197,178 |  |
|  | #8 | “随机对照试验”[常用字段:智能]OR"随机对照实验”[常用字段:智能]OR"随机对照”[常用字段:智能]OR"随机”[常用字段:智能]OR"RCT"[常用字段:智能]OR"随机对照研究"[常用字段:智能] | 1,945,548 |  |
|  | #9 | (#8)OR(#7) | 1,945,548 |  |
|  | #10 | (#9)AND(#6)AND(#3) | 0 |  |
| Clinical Trials | #1 | (Finerenone OR BAY948862)AND Diabetic Nephropathies | 5 | 18 Apr 2024 |
| Web of Science | #1 | Diabetic Nephropathies (Topic) or diabetic kidney disease(Topic) or Diabetic Kidney Diseases (Topic) or diabetes nephropathy(Topic) or diabetic nephropathy  (Topic)ordiabeticglomerulosclerosis (Topic)orDKD(Topic) or DN(Topic)orrenal diabetes (Topic)ordiabetic nephrosis(Topic) or Nephropathies,Diabetic(Topic) or  Nephropathy ,Diabetic(Topic)orkidney Disease,Diabetic (Topic)or Kidney | 254,031 | 18 Apr 2024 |
|  | #2 | Finerenone(Topic)or kerendia(Topic)or BAY94-8862(Topic) and Preprint citation  Index(Exclude-Database) | 780 |  |
|  | #3 | Randomized controlled trial(Topic)orrandom(Topic) orrandom *(Topic)orplacebo(Topic)orcontrolled clinicaltrial (Topic)ordrug therapy(Topic)orTrial(Topic) or Groups (Topic)and Preprint citation Index(Exclude-Database) | 24,330,047 |  |
|  | #4 | #1AND#2AND#3 and Preprint Citation Index (Exclude- Database) | 400 |  |
